# Supplementary material for: Chromothripsis during telomere crisis is independent of NHEJ, and consistent with a replicative origin
Source: Genome Res. 2019 May;29(5):737–49. doi: 10.1101/gr.240705.118 (PMC6499312; doi:10.1101/gr.240705.118)
Supplement: Supplemental Material [file supp_gr.240705.118_Supplemental_file_1.zip › contigs/annotated_contigs/DB73/contig.2.DB73_length_601_mean_cov_8.70549084859.docx]

**DB73_length_601_mean_cov_8.70549084859**

```
TTTTTTGTTTTTGTTTTTGTTTTTCTTCAAAATTTTTCATAGTCAGCTACAAAAGTATCTCAAATACCACATTATAGAAAAAAAAACAA
 >chr3:192466085-192466377 + E=3e-159
AAAAACACTTTCTGGTTTCATCAAATCCTTTTTACTCTTTGAAGAGTGTGATTTGGGGAAAAAAAAAACTCTATCAAGGACTATTTGTT

GAATGAGTGGTCCCTTCTAATTATTTATATTCTTAATCACCCCACAGTGAGCTTTTGGAGGAAGGTGGCCCTGTCATCTCTGGGTCTTT

CTCAAAAGTGAGAAAAAGTGATAA|AATACTTTTGTATTATATATATAATG|AGAAAATTGAATCTATTATTTTTTTCAAATACAGAAA
                                                   >chr3:192984567-192984851 - E=3e-159
AGCACAAAGAAGAAAAAAAGATGAAATCATAATCCTACCCTTCTCACCTTTTTTATGCATTACTAGTTTTTAAACTTGGGAAATACATA

GCCATTCCCAAGAAAACATTTCTAAGCTCAAGGGCCCTAAGCTTAAAGGGATTCAAAAAACTAATCTAATATTAGCTATTATTAGTAGT

AATACTATTAGTTATTGATAGTAATATATAATCCAGTTGCTGCTAGATCAAGATTTTATTGACTATGCT
```
